# Differences in Nutrition and Sensory Quality Between Cooked Soybeans, Fermented Natto, and Post-Ripening Natto

**DOI:** 10.3390/foods15020237

**Published:** 2026-01-09

**Authors:** Yuguang He, Yuanyuan Jiang, Da Li, Xue Ou, Xinyu Miao, Mubai Sun, Honghong Niu, Mei Hua, Ying Su, Jinghui Wang, Zhuo Liu

**Affiliations:** 1 Institute of Agro-Food Technology, Jilin Academy of Agricultural Sciences (Northeast Agricultural Research Center of China), Changchun 130033, China; 15044133729@163.com (Y.H.); jacken0000@163.com (D.L.); miaoxinyu@cjaas.com.cn (X.M.); 2006718850@163.com (M.S.); nhh29852@cjaas.com.cn (H.N.); huamei@cjaas.com (M.H.); suying1@163.com (Y.S.); 2 Institute of Agriculture Economy and Information, Jilin Academy of Agricultural Sciences (Northeast Agricultural Research Center of China), Changchun 130033, China; haixin-99@163.com; 3 College of Agriculture, Yanbian University, Yanji 133002, China; ouxue1002@163.com

**Keywords:** fermented foods, *Bacillus subtilis natto*, texture characteristics, flavor

## Abstract

Microbial fermentation is an important means to enhance the nutrition and functionality of food, and soybean fermentation has a long history and a wide variety of products. This study systematically compared the effects of fermentation and post-ripening processes of *Bacillus subtilis natto* JLCC513 on the nutritional components, active substances, and sensory characteristics of soybeans. The experimental results showed that, in terms of basic nutrition, fermentation led to a significant decrease in fat and reducing sugar content, followed by an initial increase and then a decrease in total protein content. In contrast, water-soluble protein continued to increase, and the total amount of free amino acids surged. The active nutritional indicators before and after soybean fermentation showed that nattokinase activity continued to increase during fermentation and post-ripening. At the same time, the number of viable bacteria decreased slightly during post-ripening. The increase in the proportion of easily absorbed aglycone-type isoflavones before and after soybean fermentation is accompanied by a sustained increase in vitamin K2 and gamma aminobutyric acid (GABA) content. In terms of sensory quality, color-difference analysis shows a decrease in brightness (L value) and an increase in redness (a value), resulting in the characteristic yellow-brown color of natto. In terms of texture characteristics, the hardness decreases, while the viscosity and elasticity are significantly enhanced. Through GC-IMS analysis of volatile aromas during soybean fermentation and post-ripening, it was found that esters (such as ethyl acetate) and pyrazines (such as 2,3-dimethylpyrazine) increased, and the product flavor shifted from grassy to fruity and nutty. In summary, natto bacteria enhance the digestibility, nutritional value, and sensory acceptance of soybeans through enzymatic hydrolysis and metabolic transformation. The post-ripening stage plays a key role in flavor maturation and further accumulation of active ingredients.

## 1. Introduction

Microbial utilization in food has a long history, dating back to before 7000 BC, when ancient civilizations used natural fermentation methods to preserve and improve food [1]. For instance, ancient Egyptians employed yeast to ferment bread [2], while ancient Chinese literature documented brewing alcohol using *Aspergillus* [3]. Today, microbial fermentation technology has become a core method in food processing [4], profoundly transforming food substrates through metabolic activities to enhance their nutritional value and sensory qualities. In today’s global society, fermented foods such as natto, yogurt, and cheese not only significantly extend food shelf life but also endow products with unique functional properties [5]. Microorganisms catalyze the transformation of substrates by secreting abundant enzyme systems that generate small-molecule peptides, vitamins, and volatile flavor compounds, thereby achieving the “bio-enhancement” of food [6]. Modern research has confirmed that probiotics in fermented foods can enhance immune function and promote nutrient absorption [7,8]. These benefits highlight the key role of microbial fermentation in the functionalization of food.

Soybeans are an important plant protein source, with protein content of 35–40%, and they contain all essential amino acids required by the human body, making them a high-quality, easy-to-digest, easily absorbed protein. Furthermore, soybeans are rich in dietary fiber, isoflavones, B vitamins, and minerals (such as calcium, iron, zinc, etc.), components that play important roles in maintaining physiological functions and enhancing immunity [9]. However, the isoflavones in soybeans mostly exist in glycosidic forms and require conversion to exert better bioactivity [10]; meanwhile, the large molecular structure of soybean protein leads to its low digestibility, and the beany flavor and bitterness limit its wide application. Traditional processing methods, such as boiling or roasting, can easily cause the loss of heat-sensitive nutrients and offer only limited improvements in sensory qualities.

As a potential probiotic, Ma et al. [11] and Sun et al. [12,13,14] found that the natto bacterium *Bacillus subtilis natto* JLCC513 can modulate the gut microbiota and intestinal barrier function in obese rats through multiple pathways, thereby contributing to weight loss. During the fermentation process, this strain can produce a variety of enzymes (including protease, amylase, and cellulase), which effectively break down macromolecules in soybeans and enhance their nutritional value. During natto fermentation, the natto bacteria multiply rapidly, and nattokinase activity increases significantly, leading to profound changes in the nutritional components of the soybeans. Protein degradation is a key step: under the action of proteases secreted by natto bacteria, soybean protein is hydrolyzed into small-molecule peptides and free amino acids, making it easier for the body to absorb and turning natto into a functional food. This comprehensively enhances the edible experience and health value of soybean products.

Post-ripening of fermented food is a crucial step in the fermentation process, which refers to the process of storing the product under specific conditions (such as specific temperature and humidity) for a period of time after the main fermentation stage, allowing it to undergo a series of slow and complex biochemical reactions, ultimately achieving the desired quality.

**Figure 1:**
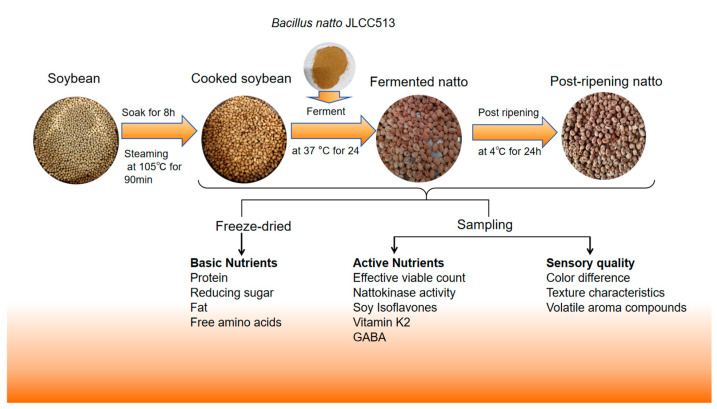
Natto and post-ripening production process and testing indicators.

Although studies have reported changes in the process of producing natto through the fermentation of soybeans with *Bacillus subtilis natto*, there is a lack of systematic analysis, especially an overall analysis of the post-ripening stage. This study utilized *Bacillus subtilis natto* JLCC513 to ferment soybeans and analyzed unfermented ripe soybeans, fermented soybeans after 24 h, and post-ripe soybeans after 24 h of ripening. The production process and testing indicators for natto and post-ripening in the experiment are shown in Figure 1. The aim was to systematically elucidate the effects of fermentation and post-ripening on the nutritional components, bioactive compounds, and sensory characteristics of soybeans, thereby filling a knowledge gap in this type of research.

## 2. Materials and Methods

### 2.1. Reagents and Strains

*Bacillus subtilis natto* JLCC513 was isolated from rice stalks in Northeast China and stored at the China General Microbial Culture and Collection Center (CGMCC, accession number: 20625). The soybeans used for fermentation are commercially available ordinary soybeans purchased from Juncheng Bean Industry (Shulan, China) in Heilongjiang Province. The natto bacterial powder is laboratory-made, with an effective live bacterial count of 100 million CUF/g. The chemical reagents were purchased from China National Pharmaceutical Group Chemical Reagent Co., Ltd. (Shanghai, China).

### 2.2. Natto Production

Referring to the method proposed by Miao et al. [15]. Soybeans soaked for 8 h were steamed at 105 °C for 90 min to obtain cooked soybeans as the control group, designated as the CS (Cooked Soybean) group. After cooling to room temperature, the soybeans were thoroughly mixed with the fermentation starter powder and incubated in a constant temperature incubator at 37 °C for 24 h to obtain natto, designated as the FN (Fermented Natto) group. After fermentation, the natto was placed in a 4 °C refrigerator for 24 h of post-ripening to obtain post-ripened natto, designated as the PRN (Post-Ripening Natto) group. Samples from the cooked soybean, natto, and post-ripened natto groups were collected for subsequent analyses.

### 2.3. Determination of Basic Nutritional Indicators During the Fermentation and Post-Ripening Process

The samples of cooked soybeans, fermented natto, and post-ripened natto were vacuum freeze-dried, pulverized, and passed through a 60-mesh sieve for subsequent analysis.

#### 2.3.1. Fat

Use the Soxhlet extraction method to determine the sample’s fat content. Dry the freeze-dried powder at 100 °C for 2 h, then place it in a dryer to cool to room temperature and weigh it. Add anhydrous ether to the Soxhlet fat extractor (Pomex, Suzhou, China) to extract the sample. After extraction, dry the sample at 100 °C for 2 h, then cool it in a dryer to a constant weight. The difference between the two masses is the fat content in the sample.

#### 2.3.2. Reducing Sugars

Determination of reducing sugars by the 3,5-dinitrosalicylic acid method. Take the freeze-dried powder, mix it with distilled water and DNS reagent in a 1:1:3 ratio, and heat it in a boiling water bath for 5 min. After cooling to room temperature, measure the absorbance at 540 nm using an enzyme-linked immunosorbent assay (Molecular Devices, Sunnyvale, CA, USA) and calculate the reducing sugar content from the glucose standard curve.

#### 2.3.3. Proteins and Water-Soluble Proteins

Measure protein content using a Kjeldahl nitrogen analyzer (Foss, Hilleroed, Denmark). Dissolve 5 g of freeze-dried powder in 50 mL of distilled water, stir thoroughly for 60 min, centrifuge at 5000 r/min for 30 min, and analyze the supernatant on a Kjeldahl nitrogen analyzer to determine water-soluble protein content.

#### 2.3.4. Free Amino Acids

The amino acid composition was analyzed using a fully automated amino acid analyzer (Sykam, Munich, Germany). The freeze-dried sample was hydrolyzed at 110 °C for 24 h in a sealed container containing 6 M HCl. After filtration and drying, the residue was dissolved in a 0.2 M sodium citrate solution (pH 2.2) and shaken in a water bath for 1 h. After filtration, it was detected in an amino acid analyzer.

### 2.4. Determination of Nutrients and Active Substances During the Fermentation and Post-Ripening Process

#### 2.4.1. Determination of Nattokinase and Effective Bacterial Count

Five grams each of cooked soybean, fermented natto, and post-ripened natto were ground and mixed with 50 mL of physiological saline. Shake and extract at 37 °C and 180 r/min for 10 min. Centrifuge at 4 °C and 10,000 r/min for 10 min. Take the supernatant to obtain the crude nattokinase enzyme solution. Take 20 μL of crude enzyme solution and spot it on a fibrin plate. Incubate at 37 °C for 16 h, measure the diameter of the transparent circle, and obtain the activity of nattokinase based on the standard curve of urokinase activity.

Determine the total bacterial count using the dilution plate method. Grind and dissolve 5 g of cooked soybeans, fermented natto, and post-cooked natto separately in 50 mL of sterile physiological saline solution. Shake and extract at 37 °C and 180 r/min for 10 min, then dilute with a 10-fold gradient. Take 100 μL of diluent and evenly spread it on the LB solid plate. Invert the plate and incubate at 37 °C for 24 h, then count the colonies.

#### 2.4.2. Soy Isoflavones

The method for analyzing isoflavones by HPLC (Thermo Fisher, Breda, The Netherlands) is based on the methods of Murphy et al. [16], Xu, and Chang [17], with slight modifications: Take 1 g of the sample into a 50 mL stoppered conical flask, dissolve it thoroughly in 3.5 mL of distilled water, add 1 mL of 0.1 mol/L HCl, and then add 5 mL of chromatographic grade acetonitrile. Shake in a gas bath shaker (Labshark, Changde, China) at room temperature (250 r/min) and extract for 2 h. After extraction, filter and take 5 mL of the filtrate. Rotate and evaporate to dryness at a temperature below 34 °C. After rotary evaporation, the residue was dissolved in 80% chromatographic-grade methanol, filtered through a 0.22 μm membrane, and placed in a 1.5 mL liquid-phase vial for later use. The chromatographic column is a Venusil MP-C18 column (5 µm, 250 × 4.6 mm i.d.); column temperature, 34 °C; detection wavelength, 262 nm; injection volume, 10 μL. Mobile phase A is H_2_O (0.3% acetic acid), mobile phase B is acetonitrile (0.3% acetic acid). The flow rate was set to 0.7 mL/min. After accurately weighing 1 g of the standard substance, dissolve it in 40 µL of dimethyl sulfoxide (DMSO) and dilute to a certain volume with 80% chromatographic-grade methanol. After being configured as a mixed standard, dilute to different concentrations, analyze by HPLC, prepare a standard curve, measure the sample, record the corresponding peak area, and calculate the content of soy isoflavones in the sample based on the standard curve.

#### 2.4.3. Vitamin K2

Isopropanol and n-hexane were mixed in a 1:2 (*v*/*v*) ratio as the extraction solution and stored. The extraction solution was mixed with the fermentation sample at a 4:1 (*v*/*v*) ratio, and the mixture was placed in an ultrasonic cleaner for 30 min to fully extract vitamin K2. The detection method of vitamin K2: HPLC was used to detect the content of vitamin K2, and the detection conditions were Shim pack Velox Biphenyl chromatographic column (2.7 μm, 4.6 mm × 100 mm); The mobile phase is methanol; Detection wavelength: 268 nm; Injection volume: 10 μL; Flow rate: 0.5 mL min^−1^.

#### 2.4.4. Gamma Aminobutyric Acid

Grind 5 g of cooked soybeans, fermented natto, and post-cooked natto separately and add them to 50 mL of physiological saline. Shake and extract at 37 °C and 180 r/min for 10 min, then centrifuge at 4 °C and 10,000 r/min for 10 min to obtain the extraction solution. Take 0.2 mL of crude enzyme solution and add 400.0 μL of borate buffer (pH 9.0), 200.0 μL of 6% phenol, and 1.0 mL of 10% sodium hypochlorite in sequence. After heating in a boiling water bath for 10 min, immediately cool in an ice bath to room temperature. After the solution turns blue-green, add 4.0 mL of 60% ethanol and measure the absorbance at 645 nm. Simultaneously, use the above method to determine the GABA standard curve. Draw a standard curve with GABA concentration as the horizontal axis and absorbance value as the vertical axis, and calculate the content of GABA in the fermentation broth.

### 2.5. Detection of Sensory Indicators During the Fermentation and Post-Ripening Process

#### 2.5.1. Color Difference

Use a colorimeter (CR-10 Plus, KONICA MINOLTA, Japan) for detection. Select 10 particles from each group, measure their L * (brightness), a * (redness), and b * (yellowness) using a colorimeter, and calculate the color difference value ΔE, ΔE = √[(ΔL*)^2^ + (Δa*)^2^ + (Δb*)^2^].

#### 2.5.2. Texture Characteristics

Using a texture analyzer (Stable Micro Systems, London, UK), the hardness, adhesiveness, resilience, cohesiveness, elasticity, gumminess, and chewiness of the samples were measured. Select 10 particles of similar size and full size for TPA extrusion testing, using a cylindrical P/36R probe with a pre-measurement speed of 5 mm/s, a mid-measurement speed of 1 mm/s, a post-measurement speed of 2 mm/s, and a compression of 75%.

#### 2.5.3. Odor Based on GC-IMS

Measurement was performed using GC-IMS (G.A.S., Dortmund, Germany), following the method of Sun et al. [18] with slight modifications. Transfer 5.0 g of fresh fermented soybeans before and after fermentation into a 20 mL headspace vial.

Headspace injection conditions: incubation temperature of 60 °C, incubation time of 15 min, oscillation speed of 500 r/min, injection needle temperature of 85 °C, injection volume of 500 μL. After each injection, purge the injection needle with high-purity nitrogen gas (purity ≥ 99.999%) for 5 min.

GC analysis conditions: The chromatographic column is a WAXX quartz capillary column (15 m × 0.53 mm, 1 μm), the column temperature is 60 °C (constant temperature mode), the carrier gas is high-purity nitrogen gas (purity ≥ 99.999%), and the carrier gas flow rate gradient is: initial flow rate is 2 mL/min, maintained for 5 min; At 5–20 min, linearly increase from 2 mL/min to 150 mL/min and maintain for 5 min, with a total running time of 25 min.

IMS analysis conditions: migration tube length of 98 mm, migration voltage of 500 V/cm, migration tube temperature of 45 °C, migration gas of high-purity nitrogen (purity ≥ 99.999%), flow rate of 150 mL/min, ionization source of tritium (3H), positive ion mode. Under the same chromatographic conditions, C4–C9 ketone (China National Pharmaceutical Group Chemical Reagent Beijing Co., Ltd., Beijing, China) was used as an external reference to calculate the retention indices (RIs) of the identified volatile compounds.

### 2.6. Data Processing

Each sample was set up in three parallel groups, and the results were expressed as mean ± standard deviation. The significance between samples was evaluated by IBM SPSS (24.0) using one-way analysis of variance (ANOVA) and the LSD test; *p* < 0.05 indicated significant differences. The results were visualized using Origin (2021) software. The VOCal (0.4.03) software was used to process the GC-IMS dataset. The Genescloud platform (https://www.genescloud.cn/) was used to generate the heatmap. The Metware Cloud platform (https://cloud.metware.cn/) was also utilized to draw a flavor wheel and a heatmap.

## 3. Results

### 3.1. Changes in Basic Nutritional Indicators During the Fermentation and Post-Ripening Process of Soybeans by Bacillus subtilis natto

#### 3.1.1. Changes in Basic Nutritional Indicators

Basic nutritional indicators can reflect the nutrient sources utilized by *Bacillus subtilis natto* during fermentation. The specific changes are summarized in Table 1. During fermentation, the fat and reducing sugar content decrease. The fat content of cooked soybeans is 17.70 g/100 g, which decreases to 16.55 g/100 g after 24 h of fermentation. At 24 h post-ripening, the fat content decreases to 15.58 g/100 g, and the reducing sugar content decreases significantly during the fermentation post-ripening process (*p* < 0.05). The total protein content significantly increased during the 24 h fermentation stage (*p* < 0.05), rising from 35.85 g/100 g to 38.23 g/100 g. After post-ripening, there was a slight decline, but it remained significantly higher than in the unfermented group (*p* < 0.05). The content of water-soluble protein showed a significant increasing trend (*p* < 0.05), reaching 4.56 g/100 g before fermentation; After 24 h of fermentation, it rapidly increased to 8.54 g/100 g; After post-ripening, it continued to increase to 9.81 g/100 g.

#### 3.1.2. Changes in Free Amino Acids

**Figure 2:**
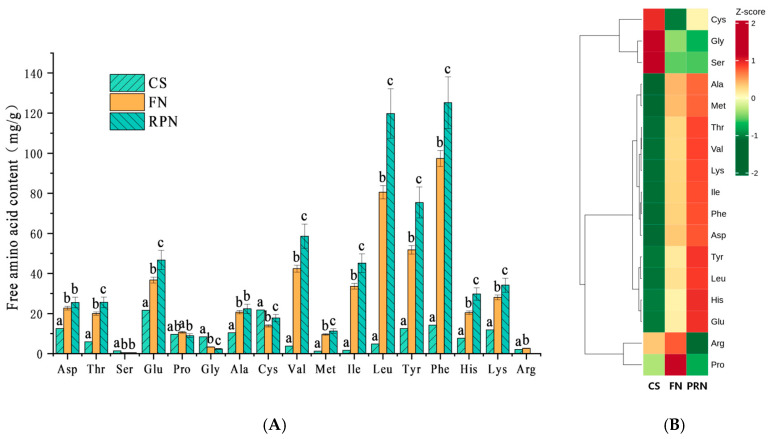
Changes in free amino acid content during the fermentation process. (**A**) Bar chart of changes in free amino acid content. (**B**) Heat map of changes in free amino acid content. The experiment was set up with three biological replicates. The values with different superscript letters in a column are significantly different (*p* < 0.05).

Further analysis of the changes in free amino acid content showed that the total free amino acid content in unfermented soybeans was 151.0 mg/100 g; After 24 h of fermentation, it soared to 427.9 mg/100 g, and further increased to 615.7 mg/100 g after post-ripening. The content of 12 amino acids continued to significantly increase (*p* < 0.05), while the content of three amino acids continued to decrease(Figure 2A). Proline and cysteine showed a trend of first increasing and then decreasing, and first decreasing and then increasing, respectively. A heatmap (Figure 2B) can more intuitively show the trend in the content of each amino acid, with 12 amino acids showing a continuous increase. In comparison, the other five amino acids exhibit different patterns of change.

### 3.2. Changes in Active Nutritional Indicators During the Fermentation and Post-Ripening Process of Soybeans by Bacillus subtilis natto

#### 3.2.1. Changes in Effective Viable Bacterial Count and Nattokinase Activity

**Figure 3:**
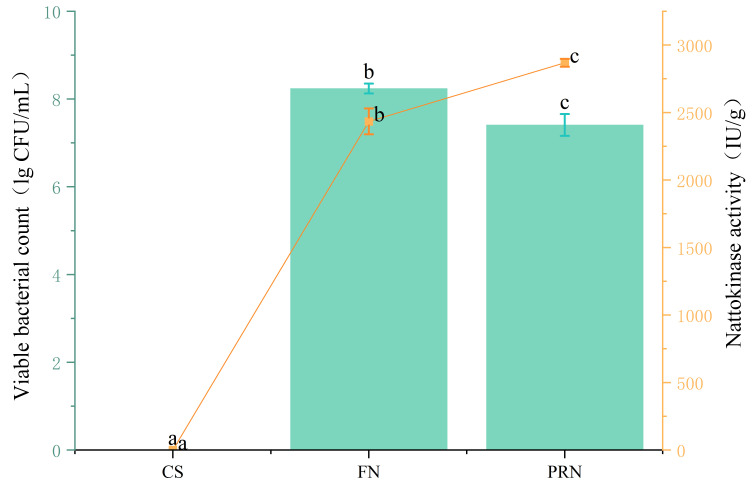
The effect of fermentation on the number of viable bacteria and nattokinase activity. The yellow line reflects the nattokinase activity across different treatments. The green columns reflects the vialbe bacteria across different treatments. The values with different superscript letters in a column are significantly different (*p* < 0.05).

The number of viable *Bacillus subtilis natto* bacteria and the activity of nattokinase can reflect the growth characteristics of this strain in the natto production process (Figure 3). The results showed that after 24 h of fermentation, nattokinase activity reached 2434.22 IU/g, and the number of live bacteria in natto reached 8.24 log CFU/mL. After post-ripening, nattokinase activity increased significantly (*p* < 0.05), but the number of live bacteria showed a slight decrease during the post-ripening stage.

#### 3.2.2. Changes in Soy Isoflavones

**Figure 4:**
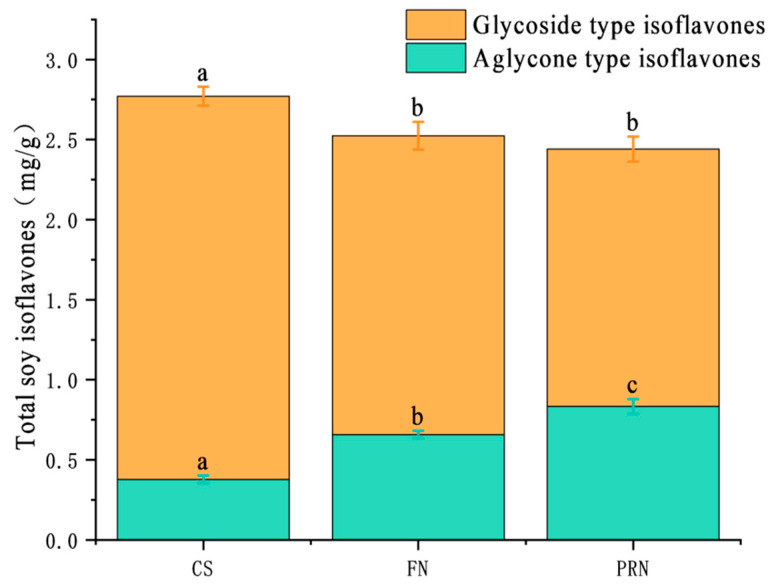
The total, glycosylated, and aglycone isoflavone contents across different treatments. The values with different superscript letters in a column are significantly different (*p* < 0.05).

The total content of soy isoflavones showed a slow decreasing trend throughout the fermentation post-ripening process (*p* < 0.05) (Figure 4). The cooked soybean group was 2.77 mg/g; after 24 h, it decreased to 2.52 mg/g; after post-ripening, it continued to decrease slightly to 2.44 mg/g. There is a significant fluctuation relationship between glycoside type isoflavones and aglycone type isoflavones. The content of glycosidic isoflavones decreased from 2.39 mg/g at the beginning to 1.87 mg/g after 24 h of fermentation, and then decreased to 1.61 mg/g after 24 h of post-ripening. During the post-ripening process, the content of aglycone-type isoflavones increased from 0.38 mg/g to 0.66 mg/g and finally reached 0.83 mg/g. The proportion of aglycones (aglycones/total isoflavones) increased from 13.7% to 34.0%.

#### 3.2.3. Changes in Vitamin K2 and Gamma Aminobutyric Acid

**Figure 5:**
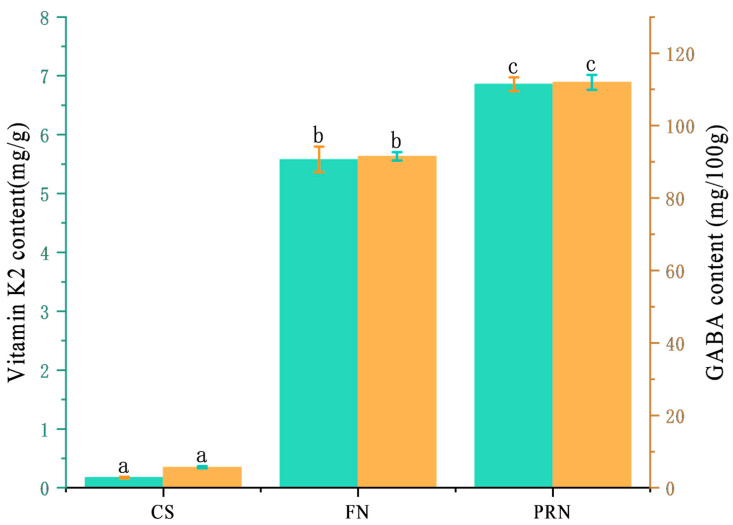
Total vitamin K2 and GABA content among different treatments. The green columns reflects the vitamin K2 content across different treatments. The yellow columns reflects the GABA content across different treatments. The values with different superscript letters in a column are significantly different (*p* < 0.05).

The vitamin K2 content continues to increase during fermentation (Figure 5), reaching only 0.18 mg/g in the 0 h sample. After 24 h of fermentation, it rapidly and significantly increases (*p* < 0.05) to 5.58 mg/g, and further increases by 22.7% after 24 h of post-ripening. The gamma-aminobutyric acid content in the 0 h sample was only 5.66 mg/100 g. During the 24 h fermentation process, the bacterial cells secreted a large amount, increasing the content to 91.54 mg/100 g. After another 24 h of post-ripening, it continued to rise to 111.96 mg/100 g.

### 3.3. Changes in Sensory Indicators During the Fermentation and Post-Ripening Process of Soybeans by Bacillus subtilis natto

#### 3.3.1. Changes in Color Difference

**Figure 6:**
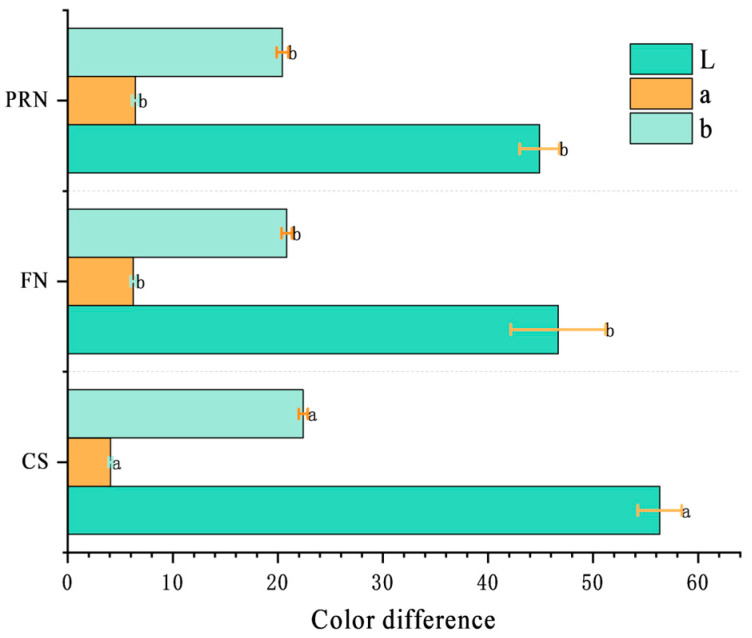
Color difference among different treatments. The values with different superscript letters in a column are significantly different (*p* < 0.05).

During fermentation and post-ripening, the surface of natto gradually changes from light yellow to off-white to a classic yellow-brown color. The L* value decreased from 58 after 0 h to 47, and then decreased to 45.59 after post-ripening; The value of a* increases continuously to 6.32, indicating that the sample has shifted from greenish to reddish; The overall change in b* value was not significant, but it also showed a significant decrease after fermentation (*p* < 0.05) (Figure 6). Compared with the post-ripening stage, the color difference changes significantly within 24 h of fermentation (*p* < 0.05), and the total color difference between cooked soybeans and post-ripe natto is ΔE = 13.1. It has exceeded the threshold recognizable by the naked eye (ΔE ≥ 3), indicating that fermentation ripening has a significant impact on the appearance and color of natto.

#### 3.3.2. Changes in Texture Characteristics

Using the TPA method to determine the seven genetic characteristics of natto, the results are presented in Table 2. The decrease in natto hardness is mainly due to the hydrolysis of soybean storage protein into small molecule peptides by various proteases secreted by natto bacteria, which destroys the protein network structure; The enhancement of adhesinerness is related to the accumulation of γ-PGA and extracellular polysaccharides, ultimately forming the typical drawing characteristics of natto; The resilence showed no significant difference between 0 and 48 h and remained around 6.3; The cohesion sharply decreased from 0.255 to 0.052 (*p* < 0.05), indicating a transition from “tight brittle” to “loose viscous” protein network, consistent with the synergistic effect of protein degradation and polysaccharide lubrication; The springiness significantly increased from 40.2% to 78% (*p* < 0.05), reflecting the enhancement of viscoelastic properties; The gumminess and chewiness decreased from 186.8 g and 75.1 g to 27.2 g and 20.9 g, respectively, with a decrease of more than 85%, indicating a shift in taste from “hard and crisp” to “soft and easy to chew”, which highly corresponds to the sensory evaluation of “melt in the mouth”.

**Table 2:** The effect of Fermentation on Texture Characteristics.

	Hardness	Adhesiveness	Resilence	Cohesion	Springiness	Gumminess	Chewiness
CS	732.82 ± 16.83 ^a^	−14.95 ± 1.46 ^a^	5.66 ± 0.96 ^a^	0.26 ± 0.01 ^a^	40.2 ± 2.56 ^a^	186.85 ± 3.44 ^a^	75.1 ± 4.81 ^a^
FN	517.22 ± 32.2 ^b^	−56.567 ± 3.16 ^b^	6.35 ± 0.9 ^a^	0.053 ± 0.02 ^b^	78.31 ± 14.72 ^b^	27.22 ± 8.54 ^b^	20.86 ± 6.31 ^b^
PRN	515.94 ± 43.06 ^b^	−59.07 ± 2.47 ^b^	6.32 ± 0.79 ^a^	0.053 ± 0.01 ^b^	78.18 ± 15.69 ^b^	27.16 ± 8.65 ^b^	18.86 ± 6.71 ^b^

The values with different superscript letters in a column are significantly different (*p* < 0.05).

#### 3.3.3. Changes in Volatile Aroma Components

**Figure 7:**
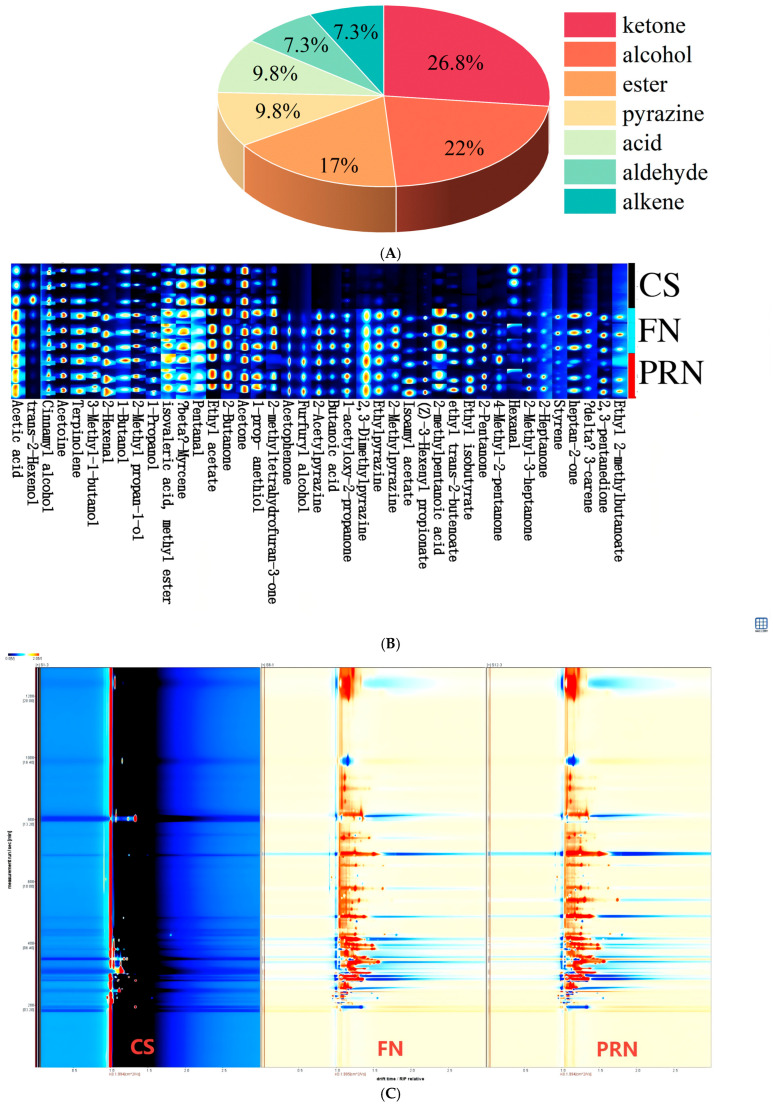

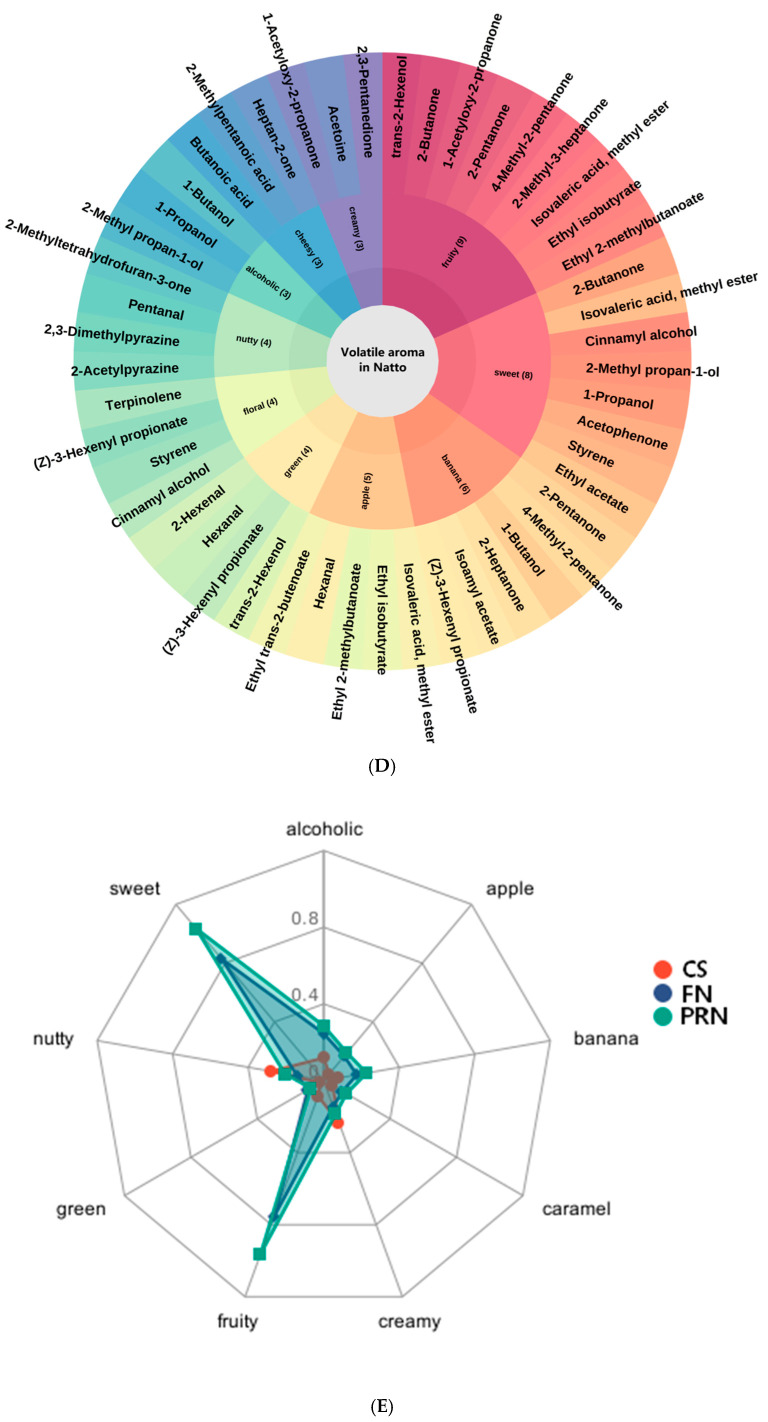

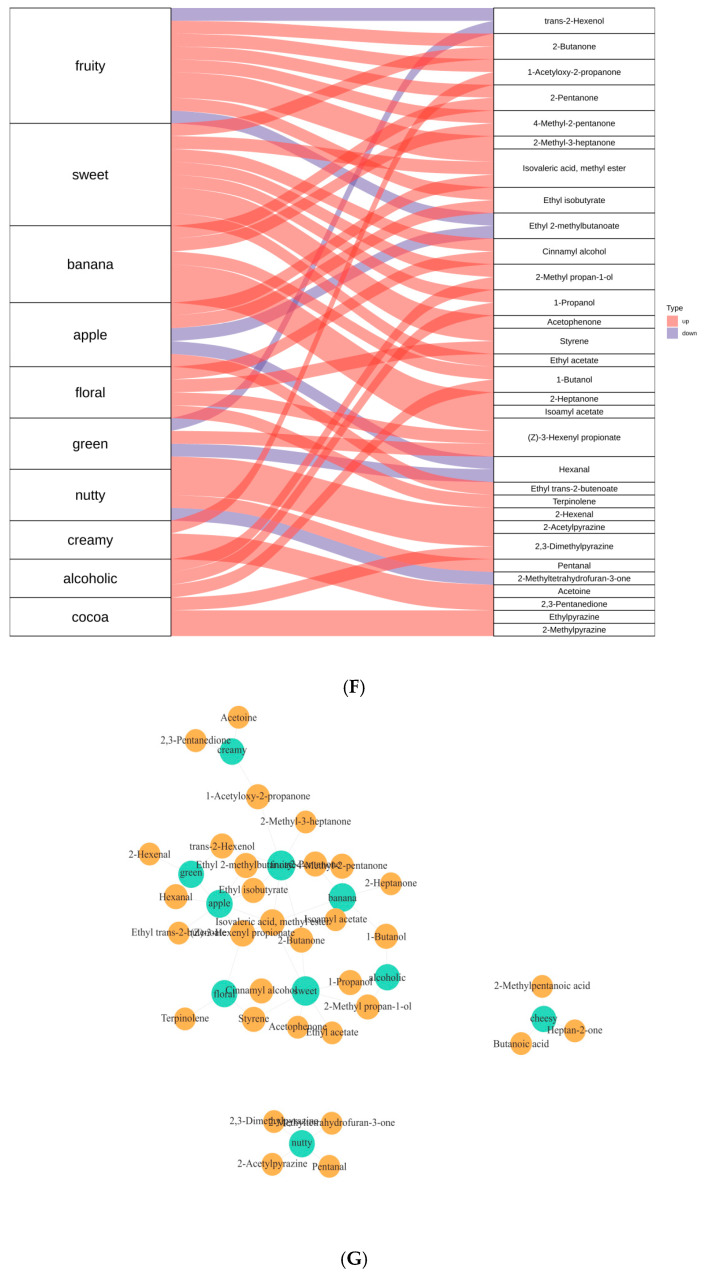
The effect of fermentation on color difference. (**A**) Percentage of each type of VOCs. (**B**) GC-IMS fingerprints. (**C**) GC-IMS spectrogram. Red indicates an increase in content, while blue indicates a decrease in content (**D**) Flavor wheel chart. (**E**) Radar chart. (**F**) Sankey diagram. (**G**) Network diagram. The experiment was set up with three biological replicates.

Analysis of changes in volatile aroma compounds during fermentation and post-ripening was conducted using SPME-GC-IMS technology. The differential GC-IMS fingerprint spectrum (Figure 7B) and spectrum (Figure 7C) display a total of 41 compounds identified during fermentation, including 11 ketones, 9 alcohols, 7 esters, 4 acids, 4 pyrazines, 3 aldehydes, and 30 alkenes. The percentage of each type of VOC (Figure 7A) shows the proportion of each substance. Throughout the fermentation and post-ripening stages, the overall levels of alcohols, ketones, and esters increased significantly, driving a shift in aroma profile from “grassy-fruity” to “soy sauce–ester” notes. Alcohols (e.g., 3-methyl-1-butanol, 1-propanol) and ketones (e.g., 2-butanone, 2,3-pentanedione) served as precursors for esterification, synergizing with acids to enhance the complexity of the overall aroma. Esters (e.g., ethyl acetate, isoamyl acetate, ethyl isobutyrate) peaked at 24 h, contributing distinct fruity flavors. Aldehydes initially increased and then decreased, a pattern linked to the balance between lipid oxidation and reduction; during fermentation, lipoxygenase-generated aldehydes were reduced to corresponding alcohols by alcohol dehydrogenase/aldehyde reductase. Acids accumulated continuously, providing both sourness and substrates for esterification. Pyrazines (e.g., 2-acetylpyrazine, 2,3-dimethylpyrazine) increased steadily during post-ripening, imparting a “roasted-nutty” aftertaste. Sulfur-containing compounds (e.g., 1-propanethiol) stabilized at high levels after 24 h, contributing to the characteristic complex flavor of natto. We can see the aroma type of each compound through the flavor wheel (Figure 7D).

Radar chart analysis (Figure 7E) revealed distinct aroma profiles among unfermented soybeans, fermented natto, and post-ripened natto. Unfermented soybeans were dominated by creamy and nutty notes, while fermented natto exhibited a gradual transition toward fruity and sweet aromas. Compared to fermented natto, the post-ripened product showed a more pronounced aroma profile: the sensory intensity of banana and apple notes increased by 20%, whereas creamy notes slightly declined, potentially due to dynamic changes in esters such as isoamyl acetate.

The Sankey diagram (Figure 7F) traced the metabolic flow of volatile organic compounds (VOCs), identifying 2-butanone and 2-heptanone as key nodes connecting “fruity” and “nutty” aromas. Meanwhile, 2,3-dimethylpyrazine directly contributed to “caramel” notes. Methyl isovalerate and ethyl acetate, as primary contributors to “banana” and “apple” aromas, respectively, increased 1.8-fold during post-ripening (*p* < 0.05).

The network diagram (Figure 7G) illustrated correlations among flavor compounds, with proximity to green nodes indicating greater contributions to specific flavors. Compounds such as 2,3-pentanedione and 2,3-dimethylpyrazine were identified as major contributors to caramel notes. Others, like 2-acetylpyrazine and 2-methyltetrahydrofuran-3-one, exhibited significant nutty characteristics. Esters such as ethyl hexanoate and active ethyl 2-methylbutyrate were closely associated with fruity notes, while acetone and 1-acetoxy-3-acetone were key components of creamy flavors. This analysis underscores how microbial fermentation transforms soybean substrates into a diverse aroma profile through coordinated biochemical pathways.

## 4. Discussion

This study systematically elucidates the multifaceted effects of the *Bacillus subtilis natto* (*B. natto*) strain JLCC513 on the nutritional, bioactive, and sensory attributes of soybeans during fermentation (0–24 h) and post-fermentation maturation (25–48 h). The results demonstrate that the impact of *B. natto* on soybeans is a complex biological process involving the synergistic actions of microbial metabolism, enzymatic catalysis, and substrate transformation, ultimately representing an “omics effect” arising from the coordinated succession of the bacterial population, enzyme systems, and the soybean matrix.

Firstly, regarding the transformation of fundamental nutrients, *B. natto* continuously consumes reducing sugars and fats in soybeans throughout the fermentation process, providing ample carbon sources and energy for its growth, reproduction, and metabolic activities. Zhou et al. [19] suggested a strong correlation between *Bacillus* species and reducing sugars, indicating that the bacteria secrete enzymes to degrade polysaccharides. Their results showed an initial increase in reducing sugar content, followed by a continuous consumption and decrease during the mid to late fermentation stages. Chen et al. [20] also confirmed the decline in reducing sugar content during *B. natto* fermentation.

The significant increase (*p* < 0.05) in total protein content during the fermentation stage, concurrent with a gradual rise in viable count, indicates substantial synthesis of bacterial protein, signifying a period of vigorous microbial growth and metabolism. The sharp and continuous increase in water-soluble protein and total free amino acid content (reaching 615.7 mg/100 g) is attributed to the continuous hydrolysis of soybean storage proteins by proteases, including nattokinase, secreted by *B. natto*. Zhang et al. [21] concluded that insoluble proteins are hydrolyzed into water-soluble proteins, which are then broken down into dipeptides or amino acids, and are further metabolized into other metabolites under the action of *B. natto*. This process not only significantly enhances the digestibility and absorption rate of proteins but also lays the foundation for natto’s umami flavor by releasing large amounts of flavor amino acids, such as aspartic acid and glutamic acid. However, the accumulation of bitter amino acids (like leucine and isoleucine) also contributes to the complexity of its flavor profile. The disappearance of arginine in the post-ripening stage suggests the activation of the arginine deiminase (ADI) pathway, which converts arginine into ornithine, citrulline, and polyamines. This may be related to the formation of amine odor precursors (such as putrescine and spermine) in natto. Furthermore, Zhou et al. [22] found that *Bacillus subtilis* efficiently utilizes arginine as an energy source for bacterial growth.

Regarding the transformation of bioactive components in soybeans, this study observed that their changes were not entirely synchronized with bacterial growth. During the post-fermentation maturation stage, the viable count decreased slightly, while levels of bioactive nutrients continued to rise. The nattokinase activity increased further in the post-fermentation stage, potentially due to the release of intracellular enzymes during bacterial autolysis or to the continued synthesis of proteases in the late fermentation phase, even though the viable count slightly declined. This indicates that using the viable count alone as an indicator of the probiotic function of natto might be insufficient, and the enzymatic activity of the matured product warrants close attention. The successful conversion of soy isoflavones from glycosidic forms to aglycones is a direct manifestation of the β-glucosidase activity of *B. natto*. Aglycone-type isoflavones can be directly absorbed through passive diffusion, increasing their bioavailability by 3–5 times, indicating that the fermentation post-ripening process significantly improves the bioavailability and physiological activity of isoflavones. Li et al. [23] similarly observed an increase in isoflavone content accompanied by elevated gene expression of β-glucosidase during natto fermentation. Vitamin K2 (MK-7) and γ-aminobutyric acid (GABA) showed a trend of continuous accumulation throughout the process. Vitamin K2 is mainly produced by the naphthoquinone synthesis pathway of natto bacteria, and its key enzymes MenA and MenD are continuously expressed in the logarithmic and stable phases. Therefore, the vitamin K2 content continues to increase during the fermentation period. Although the number of viable bacteria decreases in the post-maturation stage, the bacterial cells still maintain synthetic activity, and the intracellular storage form of vitamin K2 is released into the extracellular environment after autolysis, resulting in a continuous increase in content. Rocchi R. et al. [24] found that Vitamin K2 is synthesized by *Bacillus subtilis* during fermentation and is entirely of microbial origin; thus, it is hypothesized that the increase in Vitamin K2 is associated with the menaquinone synthesis pathway of the bacteria during the stationary phase and its release after cell autolysis. The enrichment of GABA (to 111.96 mg/100 g) is closely related to the increased content of the substrate glutamate and the activity of glutamate decarboxylase (GAD). Under the catalysis of glutamic acid decarboxylase, an alpha decarboxylation reaction occurs to generate gamma aminobutyric acid. From previous results, it is known that the free glutamic acid content during fermentation also promotes gamma-aminobutyric acid synthesis. Wang et al. [25] also confirmed that *B. natto* fermentation activates glutamate decarboxylase, catalyzing the conversion of glutamate to GABA. The increase in these two components equips natto with dual health benefits: promoting bone health (Vitamin K2) and calming nerves and regulating blood pressure (GABA).

The evolution of sensory qualities is a macroscopic manifestation of these biochemical transformations. In terms of color, the decrease in brightness (L* value) and increase in redness (a* value) resulted in a significant color difference (ΔE = 13.1), primarily associated with the Maillard reaction, polyphenol oxidation, and the formation of bacterial colonies, collectively contributing to the characteristic yellowish-brown appearance of natto. The decrease in brightness is mainly due to the growth of bacterial cells and the formation of extracellular polysaccharides, which cause micro-wrinkles on the surface, increase light-scattering losses, and lead to the deposition of brown polymers due to polyphenol oxidation during fermentation. The increase in a* represents a shift in the color of natto from greenish to reddish, which is related to the oxidation of soy isoflavones and the polymerization of phenolic substances, and is the main contribution to the typical “amber” appearance of natto. The decrease in b* value indicates that carotenoid degradation and browning reactions gradually dominate, corresponding to the sensory stage of “yellow soybeans turning into dark natto”. Kubo Y. et al. [26] reported similar findings, noting that natto hardness decreases with extended storage time. The textural results of this study similarly showed a sharp decrease in hardness, gumminess, and chewiness, alongside a significant increase in springiness and stickiness. This is presumably due to the degradation of the protein network and the production of viscous substances like γ-polyglutamic acid (γ-PGA), which collectively transform the product’s texture from the hard and crisp” texture of raw beans to the characteristically soft, sticky, and smooth texture of natto, a key determinant of consumer acceptance.

The most intricate flavor-formation process is intuitively revealed through GC-IMS technology. In the initial fermentation stage, aldehydes and alcohols dominate, imparting a “grassy” note. Zhang et al. [27] noted that alcohols, with their low odor thresholds, significantly contribute to flavor development. As fermentation progresses, the aroma evolves into a complex profile shaped by esters (e.g., ethyl acetate, isoamyl acetate), pyrazines (e.g., 2,3-dimethylpyrazine), and ketones. Ma et al. [28] similarly observed increases in alcohols, ketones, and esters during co-fermentation with *Bacillus subtilis natto* and lactobacilli. The post-fermentation maturation stage is critical for flavor refinement: further ester generation enhances “fruity” notes (e.g., banana, apple), while Chen et al. [20] highlighted that ketones—often derived from amino acid degradation or unsaturated fatty acid oxidation—contribute to consumer-preferred flavors. Concurrently, the accumulation of pyrazines provides a robust “nutty, roasted” aftertaste. Sun et al. [29] mapped dynamic changes in key volatile organic compounds (VOCs) and free amino acids in natto to KEGG pathways, revealing interconversions via the tricarboxylic acid cycle and among certain amino acids, elucidating the pathway from primary metabolites to complex flavor compounds.

Comparing the process of the transformation from mature soybeans to post mature natto during the fermentation and post-ripening stages, it can be found that the changes in the fermentation stage are due to the metabolic activity of *Bacillus subtilis natto*, which consumes the original nutrient matrix in soybeans and generates a large amount of active nutrients under the action of enzymes, which is an accumulation process. The post-ripening stage is a process of improving the quality of food. In the post-ripening stage of natto, its active nutrient content further increases, and its yellow-brown appearance deepens, making the texture and taste softer and smoother. Lyu et al. [30] also confirmed, in their study of fermented egg and dairy beverages, that the post-ripening stage can enhance the nutritional quality of fermented foods. The change in aroma components during the post-ripening stage is the most crucial, shifting from the “green grass fruit aroma” at the end of fermentation to the “sauce ester aroma”, making the flavor of natto more mellow. Wu et al. [31] found that the activity of microbial colonies during the post-ripening stage is closely related to the formation of product flavor. Deng et al. [32] also studied changes in the aroma of Jinhua ham during post-ripening. Weng et al. [33] also studied the evolution of aroma in several fermented teas during the post-ripening stage. This indicates that the post-ripening stage is an important stage for aroma transformation and for improving the flavor quality of fermented foods.

In summary, *Bacillus subtilis natto* fermentation systematically upgrades soybean quality. This process transforms nutritionally rich but hard-to-digest, bland-flavored soybeans into a health-promoting food with enhanced protein bioavailability, diverse functional components, and unique texture and flavor profiles. This study preliminarily elucidates the dynamics of nutritional and sensory quality changes during fermentation, laying a foundation for understanding the underlying mechanisms. Future research could integrate multi-omics approaches (e.g., transcriptomics, proteomics) with enzyme kinetics and metabolic flux analysis to dissect molecular-level transformation pathways and regulatory mechanisms. The findings provide a theoretical basis for selecting superior starter cultures, developing high-activity natto products, and establishing a more scientific, comprehensive quality evaluation system for natto.

## 5. Conclusions

This study demonstrates that *Bacillus subtilis natto* JLCC513 can systematically convert soybeans into a food product with high protein bioavailability, rich in various functional components, and a unique flavor during fermentation (0–24 h) and post-ripening (25–48 h).

During the fermentation stage, the mycelium rapidly consumes reducing sugars and fats as carbon and energy sources through metabolic activities, promoting mycelial protein synthesis and significantly increasing the total protein content. The secreted proteases, such as nattokinase, hydrolyze soybean storage proteins into water-soluble proteins and free amino acids, thereby greatly improving the digestion and absorption of proteins and releasing umami substances, such as glutamic acid, and bitter amino acids, such as leucine, enriching the flavor profile. The degradation of protein networks and the production of viscous substances, such as γ-polyglutamic acid, give it a soft, smooth texture. The flavor at this stage transitions from a single-bean aroma to a dominant, creamy, nutty aroma.

During the post-ripening stage, the number of viable bacteria decreases slightly, while the active components continue to accumulate. The activity of nattokinase is further. Vitamin K2 (MK-7) is continuously produced through the naphthoquinone synthesis pathway of the bacteria, and γ-aminobutyric acid (GABA) is enriched under the catalysis of glutamate decarboxylase. These components endow natto with various health potentials. During the post-ripening period, the color and taste changes are not significant, but the aroma transitions from “green grass fruit aroma” to “sauce ester aroma”.

This study preliminarily elucidated the dynamic changes in nutrition and sensory quality during fermentation, providing a theoretical basis for subsequent analyses of molecular mechanisms, strain optimization, and the establishment of a comprehensive evaluation system for natto quality using multi-omics technology.

## Figures and Tables

**Table 1:** Changes in basic nutritional indicators of soybeans before and after fermentation by natto bacteria.

	Fat (g/100 g)	Reduced Sugar (g/100 g)	Protein (g/100 g)	Water Soluble Protein (g/100 g)
CS	17.7 ± 0.15 ^a^	1.46 ± 0.09 ^a^	35.85 ± 0.48 ^a^	4.56 ± 0.21 ^a^
FN	16.55 ± 0.31 ^b^	1.24 ± 0.06 ^b^	38.23 ± 0.31 ^b^	8.54 ± 0.22 ^b^
PRN	15.58 ± 0.28 ^c^	0.92 ± 0.04 ^c^	37.28 ± 0.35 ^c^	9.81 ± 0.28 ^c^

The values with different superscript letters in a column are significantly different (*p* < 0.05).

## Data Availability

The original contributions presented in this study are included in the article. Further inquiries can be directed to the corresponding authors.
